# Striatal dopamine transporter imaging in Parkinson’s disease drug-naïve patients: focus on sexual dysfunction

**DOI:** 10.1007/s10072-022-06050-7

**Published:** 2022-04-06

**Authors:** Elena Contaldi, Luca Magistrelli, Silvia Gallo, Cristoforo Comi

**Affiliations:** 1 Department of Translational Medicine, Movement Disorders Centre, “Maggiore Della Carità” University Hospital, University of Piemonte Orientale, Corso Mazzini 18, 28100 Novara, Italy; 2 PhD Program in Medical Sciences and Biotechnology, University of Piemonte Orientale, Novara, Italy; 3 PhD Program in Clinical and Experimental Medicine and Medical Humanities, University of Insubria, Varese, Italy; 4 Department of Translational Medicine, Neurology Unit, S. Andrea Hospital, University of Piemonte Orientale, Vercelli, Italy

**Keywords:** Sexual dysfunction, 123-I-FP-CIT SPECT, Parkinson’s disease, Drug-naïve

## Abstract

**Introduction:**

Dopamine is involved in sexual behavior, but dopaminergic imaging studies establishing the relationship between nigrostriatal dopaminergic degeneration and sexual dysfunction (SD) in Parkinson’s disease (PD) are lacking.

**Methods:**

We retrospectively analyzed clinical and ^123^I-FP-CIT SPECT data of 43 drug-naïve PD patients. Based on the sexual function domain of the Non-Motor Symptoms Scale (NMSS), we identified 23 patients with sexual concerns (WSC), reporting a score ≥ 2 due to hyposexuality, and 20 patients without sexual concerns (NoSC). Dopamine transporter (DAT) uptake was assessed through semi-quantitative analysis in the most and least affected putamen (maP, laP), and most and least affected caudate (maC, laC). Total putamen-to-caudate ratio and total striatal binding ratio (tSBR) were also quantified.

**Results:**

WSC and NoSC had similar demographic and disease-related characteristics. WSC displayed lower uptake values in maC (*p* = 0.016), maP (*p* = 0.004), laC (*p* = 0.019), laP (*p* = 0.009), and tSBR (*p* = 0.006). Pearson correlation analysis revealed, in the WSC group, moderate inverse correlations between the log-transformed SD scores and the uptake in maP (*r* =  − 0.473, *p* = 0.023), maC (*r* =  − 0.428, *p* = 0.042), laP (*r* = -0.437, *p* = 0.037), and tSBR (*r* =  − 0.460, *p* = 0.027). After controlling in a two-way ANCOVA model for age and sex, between-group differences,between WSC and NoSC remained statistically significant only for dopaminergic denervation in maP [*F*(1,38) = 7.478, *p* = 0.009)], laP [*F*(1,38) = 4.684, *p* = 0.037)], and tSBR [*F*(1,38) = 5.069, *p* = 0.030].

**Conclusion:**

To the best of our knowledge, this is the first study reporting the relationship between the severity of SD and specific patterns of nigrostriatal dopaminergic denervation (especially involving both putamina) in newly diagnosed drug-naïve PD patients.

## Introduction

Parkinson’s disease (PD) is one of the most common neurodegenerative diseases, with more than 10 million people affected worldwide. The pathological hallmark of the disease is represented by intraneuronal α-synuclein-positive inclusions called Lewy bodies and loss of dopaminergic neurons in the substantia nigra pars compact (SNc), the dorsal motor nucleus of the vagal nerve, the locus coeruleus, the pedunculopontine nucleus, and the nucleus basalis of Meynert [1]. The widespread distribution of Lewy bodies reflects the complexity and heterogeneity of the clinical picture, which is burdened by motor symptoms, including bradykinesia, rigidity, tremor and postural instability, and a wide array of non-motor symptoms [2]. Among the most disabling non-motor features of PD, sexual dysfunction (SD) is very common yet poorly understood and underinvestigated [3]. Basson described the sexual concerns (SC) of 25 people with PD, reporting altered sexual function (erectile dysfunction, vaginismus, difficulty in reaching orgasm, internal tremor during arousal), and lower self-esteem [4]. When considering the prevalence of these symptoms compared with healthy controls, one study [5] found that SD was more frequently reported in male PD patients, with age as the main predictor, and similar results were observed in women with PD [6]. Regarding factors associated with SD occurrence, a recent cross-sectional multicenter study [7] found in a cohort of 105 patients with early-onset PD a significant relationship with sex, higher depression scores, and urinary dysfunction. Even though a multinational survey among neurologists revealed a lack of discussing SD, especially for female patients [8], current literature is emphasizing the importance of addressing and solving sexual issues: it was demonstrated in a longitudinal study involving a large cohort of PD patients that sexual activity is associated with better motor and non-motor outcomes and better quality of life [9].

It is known that dopamine plays a key role in sexual behavior [10]. In the medial preoptic area, which is the main integrative site for male sexual behavior, the release of dopamine promotes copulation, genital reflex, and sexual motivation [11, 12]. The stimulation of dopamine receptors (mainly of the D2 to D4 subtype) in the paraventricular nucleus induces the release of oxytocin and exerts pro-erectile effects by increasing extra-cellular dopamine in the nucleus accumbens in male rats [13]. Furthermore, dopamine reduces the release of prolactin, which is known to interfere with libido [14].

A wide variety of PD non-motor symptoms has been proved to display an underlying dopaminergic basis, as also confirmed by imaging techniques with dopamine-based radioligands [15, 16]. On the other hand, conflicting results reporting the lack of association between dopamine transporter (DAT) loss and the burden of several non-motor symptoms in PD have been observed as well [17, 18]. In this context, the relationship between nigrostriatal dopaminergic degeneration and the severity of SD in PD has not yet been fully elucidated. To this aim, the present study evaluated ^123^I-FP-CIT single-photon emission computed tomography (SPECT) data in relation to SD in a cohort of newly-diagnosed drug-naïve PD patients.

## Methods

### Study participants

This research was carried out according to the ethical guidelines of the local Ethics Committee and all patients gave their written informed consent (CE 65/16). In this retrospective cross-sectional study, we analyzed an electronic database containing clinical and SPECT data of drug-naïve PD patients referring to our Movement Disorders outpatient clinic between January 1, 2012, and January 1, 2020. We considered as inclusion criteria a diagnosis of PD according to the United Kingdom Parkinson’s Disease Society Brain Bank Diagnostic Criteria [19], and no history of current or previous therapy with antiparkinsonian agents. Exclusion criteria were brain abnormalities on magnetic resonance imaging, the occurrence of atypical signs or symptoms in conflict with a diagnosis of idiopathic PD during subsequent clinical re-assessments, and a history of pelvic surgery, dementia, or severe depression. At the time of baseline visit, neurological examination was performed by neurologists with experience in movement disorders, and motor symptoms were assessed using the Unified Parkinson’s Disease Rating Scale (UPDRS) part III and the Hoehn and Yahr (HY) scale [20, 21]. All the patients undergoing the SPECT study were not taking central nervous system-acting drugs potentially interfering with the analysis [22] and were in the drug-naïve condition concerning antiparkinsonian treatment. Other relevant demographic and clinical data were collected, including sex, age, disease duration, side of onset of motor symptoms, clinical phenotype, a history of hyposmia or anosmia, concurrent therapy, and general medical records. REM sleep behavior disorder (RBD) was assessed through the RBD screening questionnaire (RBDSQ), which is a reliable tool for detecting RBD in PD patients [23, 24].

From our initial cohort of 118 consecutive PD patients with ^123^I-FP-CIT SPECT analysis available, 61 PD subjects also underwent within 3 months a detailed neuropsychological evaluation through the Mini-Mental State Examination (MMSE) [25] and the Addenbrooke’s Cognitive Examination-revised (ACE-R) [26]. Depression, anxiety, and non-motor symptoms were respectively assessed through the Beck Depression Inventory (BDI)-II [27], the Zung Self-rating Anxiety Scale [28], and the Non-Motor Symptoms Scale (NMSS) [29]. Among the responders of the NMSS sexual function items (43 out of 61, response rate = 70.5%), we identified the patients with sexual concerns (WSC), who reported a global score ≥ 2 due to hypoactive sexual desire and/or function, and the patients without sexual concerns (NoSC). The remaining 18 patients left this subscore incomplete or unanswered.

### Semi-quantitative dopamine transporter SPECT analysis

Brain SPECTs were acquired using standard procedures: 140–180 MBq of ^123^I-FP-CIT (DaTSCAN®, GE Healthcare Ltd, Little Chalfont, UK) were injected intravenously 40–60 minafter administration of KClO4 400 mg to block free iodine uptake into the thyroid. The patients were imaged 4 h post-injection using a dual-head gamma-camera (Philips Axis) equipped with low-energy high-resolution parallel hole collimators. To optimize spatial resolution and reduce rotation radius, the patients were positioned to include the whole neurocranium in the field of view with the exclusion of salivary glands. One hundred twenty views were acquired using a step-and-shoot protocol at 3° interval, and images were visually inspected by experienced nuclear physicians. Motion artifacts were excluded and the quality of images was considered suitable for diagnostic purposes. To meet Basal Ganglia (BasGan) Matching Tool software requirements [30, 31], all the sets were reconstructed using a Butterworth filter (order = 7.0, cut-off = 0.45) and corrected for attenuation using Chang algorithm (*μ* = 0.10 cm − 1). Transaxial images were reoriented on the orbitomeatal line. The semi-quantitative analysis was performed automatically by positioning a 3D region of interest (ROI) template, including an occipital ROI for background evaluation. To obtain specific striatal binding ratios (SBR), occipital background binding was subtracted from the putamen and caudate nucleus uptake of the most and least affected hemisphere, according to motor symptoms, following the formula: SBR = (caudate or putamen ROI count density − occipital ROI count density) / occipital ROI count density. Data from the analyzed nuclei were then compared to a reference database [30] to obtain age-adjusted values for each basal ganglion. DAT uptake in the most and least affected putamen (maP, laP), and most and least affected caudate (maC, laC) was assessed. The total putamen-to-caudate ratio (tP/C) and total striatal binding ratio (tSBR) were respectively calculated by averaging the putamen-to-caudate ratio from each hemisphere and the uptake measures in the four analyzed striatal areas.

### Statistical analyses

Variables were expressed as counts (percentages) when categorical and as mean (± standard deviation) when continuous. The normality of data distribution was assessed both visually and using the Shapiro–Wilk test, and parametric or non-parametric tests were used as appropriate. Log-transformation for NMSS sexual function scores was performed, as we observed skewed distribution of this variable. The relationship between log-transformed data and regional DAT availability was then investigated through Pearson correlation analysis. Categorical variables were compared using Fisher’s exact test, whereas the *T*-test for independent samples or the Mann–Whitney test were used to explore differences in means or medians between groups. We then controlled in a two-way analysis of covariance (ANCOVA) model for the effect of remaining potential confounders. When adjusting for covariates, assumptions for homogeneity of regression slopes and the absence of interaction between each covariate, and factors were tested. The significance level was set to *p* < 0.05. All the analyses were performed using SPSS Version 25 (IBM Corporation, Armonk, USA).

## Results

**Fig. 1:**
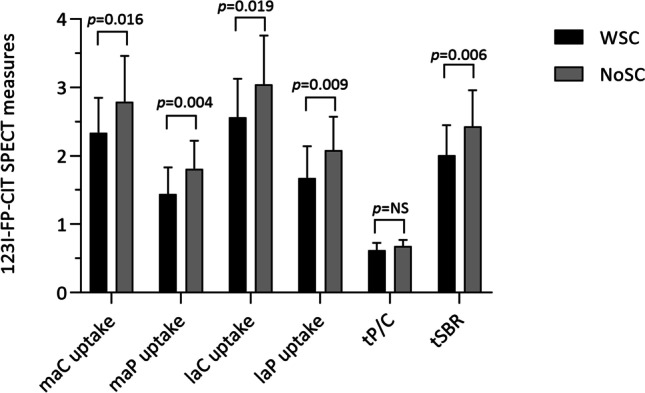
Differences in ^123^I-FP-CIT SPECT measurements (maC, most affected caudate; maP, most affect putamen; laC, least affected caudate; laP, least affected putamen; tP/C, total putamen-to-caudate ratio; tSBR, total striatal binding ratio) in PD patients with (WSC) and without (NoSC) sexual concerns

**Fig. 2:**
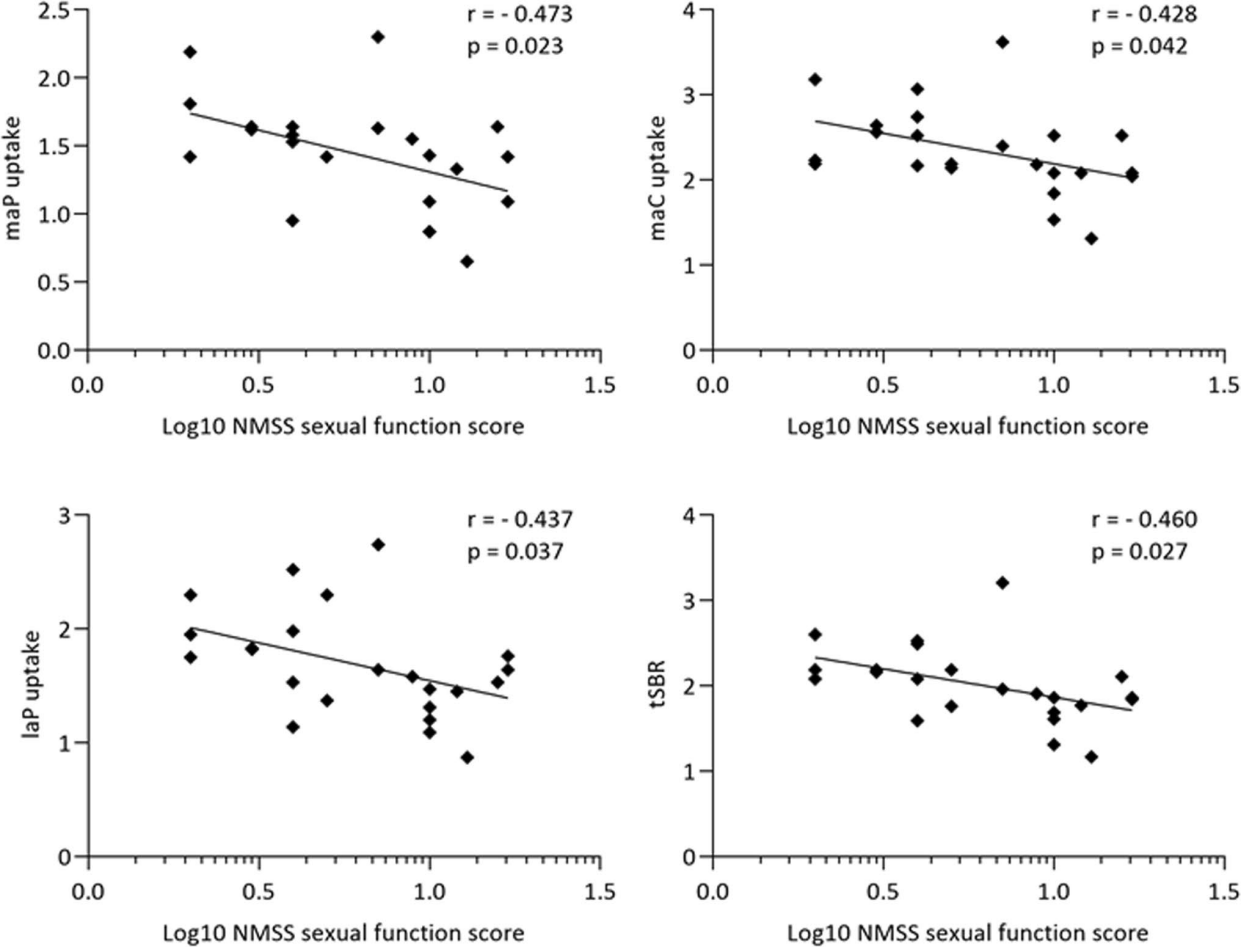
Scatterplot graphs representing significant Pearson correlation analysis between ^123^I-FP-CIT SPECT measurements (maP, most affect putamen; maC, most affected caudate; laP, least affected putamen; tSBR, total striatal binding ratio) and log-transformed NMSS sexual function scores

This study involved 43 PD patients, 15 females and 28 males, mean age 65.40 ± 8.32 years, with a mean disease duration of 1.49 ± 0.83 years and mean UPDRS part III score of 11.63 ± 4.01. According to the NMSS sexual function scores, we identified 23 WSC and 20 NoSC. Group comparisons between WSC and NoSC of demographic and clinical characteristics, neuropsychiatric measures, medical conditions, concurrent therapy, and ^123^I-FP-CIT SPECT data, are shown in Table 1. We found that both groups displayed similar demographic and disease-related characteristics, even though WSC consisted of older and predominantly male patients compared with NoSC. Regarding DAT SPECT analysis, WSC patients had significantly lower uptake values in the maC (*p* = 0.016), maP (*p* = 0.004), laC (*p* = 0.019), laP (p = 0.009), and tSBR (*p* = 0.006) (see Fig. 1). Furthermore, in the WSC group, Pearson correlation analysis revealed moderate inverse correlations between the log-transformed NMSS sexual function scores and the uptake in the maP (*r* =  − 0.473, *p* = 0.023), maC (*r* =  − 0.428, *p* = 0.042), laP (*r* =  − 0.437, *p* = 0.037), and tSBR (*r* =  − 0.460, *p* = 0.027) (see Fig. 2). After controlling in a two-way ANCOVA model for potential confounders (i.e., age and sex), between-group differences between WSC and NoSC remained statistically significant only for the uptake in the maP [*F*(1,38) = 7.478, *p* = 0.009)], laP [*F*(1,38) = 4.684, *p* = 0.037)], and tSBR [*F*(1,38) = 5.069, *p* = 0.030], see Table 2.

**Table 1:** Group comparisons between WSC and NoSC of demographic and clinical characteristics, neuropsychiatric measures, medical conditions, concurrent therapy, and ^123^I-FP-CIT SPECT data. Variables were expressed as counts (percentages) when categorical and as mean (± standard deviation) when continuous

	**WSC (*n* = 23)**	**NoSC (*n* = 20)**	***p*-value**
Age	67.13 (9.05)	63.40 (7.09)	0.144
Sex, M/F	17/6	11/9	0.219
Tremor dominant phenotype, *n* (%)	16 (69.6)	15 (75)	0.745
Right side of onset, *n* (%)	15 (65.2)	10 (50)	0.365
Disease duration, years	1.54 (0.91)	1.43 (0.74)	0.829
UPDRS-III score	11.78 (4.17)	11.45 (4.09)	0.794
Hoehn and Yahr stage			
Stage 1–1.5, *n* (%)	15 (65.2)	16 (80)	0.327
Stage 2–2.5, *n* (%)	8 (34.8)	4 (20)	0.327
RBD, *n* (%)	12 (52.2)	7 (35)	0.359
Hyposmia, *n* (%)	13 (56.5)	10 (50)	0.547
Sexual function score NMSS	7.65 (4.87)	0	< *0.0001*
Gastrointestinal score NMSS	3.22 (3.69)	3.20 (5.79)	0.377
Urinary score NMSS	5.90 (8.17)	5.61 (8.40)	0.806
MMSE score	27.56 (1.94)	27.79 (1.82)	0.711
ACE-R score	89.69 (6.81)	91 (7.01)	0.625
BDI-II score	11.22 (8.78)	9.74 (8.99)	0.310
Zung Anxiety Scale Score	36.28 (9.72)	33.41 (10.27)	0.296
Use of antidepressants, *n* (%)	4 (17.4)	3 (15)	1.0
Cardiovascular disease, *n* (%)	3 (13)	3 (15)	1.0
History of diabetes, *n* (%)	2 (8.7)	0	0.491
History of hypertension, *n* (%)	12 (52.1)	7 (35)	0.359
History of dyslipidemia, *n* (%)	1 (4.3)	3 (15)	0.323
Use of thiazides, *n* (%)	1 (4.3)	1 (5)	1.0
Use of beta-blockers, *n* (%)	2 (8.7)	2 (10)	1.0
maC uptake	2.34 (0.51)	2.79 (0.67)	*0.016*
maP uptake	1.44 (0.39)	1.81 (0.41)	*0.004*
laC uptake	2.56 (0.57)	3.04 (0.72)	*0.019*
laP uptake	1.68 (0.46)	2.08 (0.49)	*0.009*
tP/C	0.62 (0.11)	0.68 (0.09)	0.078
tSBR	2.01 (0.44)	2.43 (0.53)	*0.006*

*WSC* patients with sexual concerns, *NoSC* patients without sexual concerns, *UPDRS-III* Unified Parkinson’s Disease Rating Scale-III, *RBD* REM sleep behavior disorder, *NMSS* Non-Motor Symptoms Scale, *MMSE* Mini-Mental State Examination, *ACE-R* Addenbrooke’s Cognitive Examination Revised, *BDI-II* Beck Depression Inventory-II, *maC* most affected caudate, *maP* most affected putamen, *laC* least affected caudate, *laP* least affected putamen; *tP/C* total putamen to caudate ratio, *tSBR* total striatal binding ratio. Significant *p*-values are in italics

**Table 2:** Two-way ANCOVA for ^123^I-FP-CIT SPECT data adding age and sex as covariates in the model

	***F***	***p***	***Partial eta squared***
maC uptake			
Age	*F*(1,38) = 0.988	0.327	0.025
Sex	*F*(1,38) = 2.793	0.103	0.068
Groups	*F*(1,38) = 3.755	0.060	0.090
maP uptake			
Age	*F*(1,38) = 2.487	0.123	0.061
Sex	*F*(1,38) = 0.003	0.954	0.000089
Groups	*F*(1,38) = 7.478	*0.009*	0.164
laC uptake			
Age	*F*(1,38) = 0.00028	0.987	0.000008
Sex	*F*(1,38) = 0.800	0.377	0.021
Groups	*F*(1,38) = 2.885	0.098	0.071
laP uptake			
Age	*F*(1,38) = 0.008	0.929	0.00021
Sex	*F*(1,38) = 0.001	0.973	0.00003
Groups	*F*(1,38) = 4.684	*0.037*	0.110
tSBR			
Age	*F*(1,38) = 0.412	0.525	0.011
Sex	*F*(1,38) = 0.663	0.421	0.017
Groups	*F*(1,38) = 5.069	*0.030*	0.118

*maC* most affected caudate, *maP* most affected putamen,; *laC* least affected caudate, *laP* least affected putamen, *tSBR* total striatal binding ratio. Significant *p*-values are in italics

## Discussion

This research found that drug-naïve PD patients WSC have lower DAT availability compared with NoSC and exhibit moderate inverse correlations between SD severity and DAT uptake in the maP, maC, laP, and tSBR. However, after controlling for relevant confounding factors, significantly reduced uptake was confirmed only in maP, laP, and tSBR. To the best of our knowledge, this is the first study reporting the relationship between specific patterns of nigrostriatal dopaminergic denervation and the severity of sexual function reduction in newly diagnosed drug-naïve PD patients, suggesting that reduced striatal DAT uptake may be an early mechanism involved in this disabling non-motor symptom.

SD is a complex phenomenon, in which organic causes, as well as environmental and psychological factors, are involved. Although it is increasingly being recognized as a key component of medical assessment since it may markedly influence patients’ quality of life [9], a deeper knowledge of the implicated neuronal circuits is still lacking. It is known that subcortical structures are interconnected with regions responsible for the regulation of autonomic mechanisms, emotions, and somatosensory processing involved in sexual activity. In this context, our study provides intriguing evidence that highlights the role of defective dopaminergic uptake of both maP and laP in PD-related SD. Several lines of research could support this finding: firstly, it was shown in monkeys that the stimulation of the dorsal putamen and anterior region of the internal capsule was able to induce erections [32]. Secondly, fMRI investigations exploring neural substrates of sexual arousal reported the activation of putamen and globus pallidus, which were involved in the physiological and rewarding aspects of the sexual stimuli [33]. Interestingly, a recent study by Votinov et al. [34] assessed brain structure changes associated with sexual orientation, and the putamen emerged as the only region with increased gray matter volume in homosexual versus heterosexual individuals, thus expanding the role of putamen in the complex and various aspects of human sexuality.

On the other hand, when examining PD non-motor features such as anxiety, depression, and apathy, previous literature has emphasized the role of the caudate nucleus. Erro et al. [35] found in 34 untreated PD patients a significant correlation between increased anxiety severity and decreased DAT availability in the right caudate. Vriend et al. [36] reported in 100 PD patients that depressive symptoms were related to lower DAT binding in the right caudate nucleus, whereas decreased DAT binding in the right putamen was associated with worse motor symptoms. Regarding apathy, another study [37] found that the dysfunction of dopaminergic innervation, particularly in the right caudate, contributed to the development of apathy in PD.

Even though we found decreased DAT availability in the maC (*p* = 0.016) and laC (*p* = 0.019) of WSC patients, this finding was not supported in the ANCOVA model after controlling for age and sex. Indeed, to exclude potential confounders in assessing SD and its relationship with nigrostriatal dopaminergic denervation patterns, several factors were taken into careful consideration. Firstly, a history of cardiovascular disease, diabetes, hypertension, and dyslipidemia, and the use of thiazides, beta-blockers, and antidepressants, are notably associated with an increased risk of SD [10]. However, there were no relevant differences between groups in these variables of interest, thus reasonably excluding their potential interference in the assessment of SD. Secondly, demographic, motor characteristics (tremor dominant versus rigid akinetic phenotype), as well as other non-motor symptoms (i.e., hyposmia, REM sleep behavior disorder, anxiety-depressive disorder, gastrointestinal, urinary, and cognitive impairment), can all be associated with altered DAT SPECT denervation patterns [15, 16, 38, 39]. Nonetheless, WSC and NoSC groups were largely comparable regarding relevant disease-related characteristics, and we controlled for remaining significant confounders in the ANCOVA analysis. Another strength of the present study is represented by the accurate selection of a cohort of PD drug-naïve patients, as it is well established that treatment with dopaminergic therapy may increase sexual desire, sometimes even leading to compulsive behaviors [40].

We acknowledge that the use of a retrospective design and the relatively small sample size represent limitations of the present study. Furthermore, the reduced number of WSC patients prevented the analysis of different DAT SPECT patterns associated with NMSS sexual domain subitems, i.e., loss of libido and sexual difficulties, and an extensive evaluation of SD through detailed rating scales is lacking as well. Another limitation is represented by the time range between DAT SPECT and NMSS evaluation, even though we can assume that these symptoms did not considerably change in a few months. Finally, we cannot exclude that non-dopaminergic dysfunction (including degeneration of serotoninergic pathways), though not investigated in the present study, may provide an important contribution to the development of SD [41]. Despite these drawbacks, this is the first study, to the best of our knowledge, assessing the relationship between nigrostriatal dopaminergic denervation and the severity of SD in PD.

## Conclusions

In conclusion, in newly diagnosed drug-naïve PD patients, reduced striatal DAT availability (especially in the putamina) may be involved in SD. Nonetheless, larger cohorts and longitudinal analyses will better evaluate the association between specific patterns of DAT loss and SD. We also suggest that future studies should explore the optimization of dopamine replacement therapy in PD patients as a suitable approach to improve this disabling and often underestimated problem.

## Data Availability

Anonymized data can be obtained upon reasonable request from qualified researchers.

## References

[1] Braak H, Tredici KD, Rüb U (2003). Staging of brain pathology related to sporadic Parkinson’s disease. Neurobiol Aging.

[2] Obeso JA, Stamelou M, Goetz CG (2017). Past, present, and future of Parkinson’s disease: a special essay on the 200th Anniversary of the Shaking Palsy. Mov Disord.

[3] van Hees PJM, van der Plas AA, van Ek GF (2017). Discussing sexuality with patients with Parkinson’s disease: a survey among Dutch neurologists. J Neural Transm.

[4] Basson R (1996). Sexuality and Parkinson’s disease. Parkinsonism Relat Disord.

[5] Shalash A, Hamid E, Elrassas H (2020). Sexual dysfunction in male patients with Parkinson’s disease: related factors and impact on quality of life. Neurol Sci.

[6] Varanda S, Ribeiro da Silva J, Costa AS (2016). Sexual dysfunction in women with Parkinson’s disease. Mov Disord.

[7] Vela-Desojo L, Urso D, Kurtis-Urra M (2020). Sexual dysfunction in early-onset parkinson’s disease: a cross-sectional, multicenter study. J Parkinsons Dis.

[8] de Rooy FBB, Buhmann C, Schönwald B (2019). Discussing sexuality with Parkinson’s disease patients: a multinational survey among neurologists. J Neural Transm (Vienna).

[9] Picillo M, Palladino R, Erro R (2019). The PRIAMO study: active sexual life is associated with better motor and non-motor outcomes in men with early Parkinson’s disease. Eur J Neurol.

[10] Meco G, Rubino A, Caravona N, Valente M (2008). Sexual dysfunction in Parkinson’s disease. Parkinsonism Relat Disord.

[11] Dominguez J, Hull E (2005). Dopamine, the medial preoptic area, and male sexual behavior. Physiol Behav.

[12] Hull EM, Dominguez JM (2006). Getting his act together: roles of glutamate, nitric oxide, and dopamine in the medial preoptic area. Brain Res.

[13] Succu S, Sanna F, Melis T (2007). Stimulation of dopamine receptors in the paraventricular nucleus of the hypothalamus of male rats induces penile erection and increases extra-cellular dopamine in the nucleus accumbens: involvement of central oxytocin. Neuropharmacology.

[14] Krüger THC, Hartmann U, Schedlowski M (2005). Prolactinergic and dopaminergic mechanisms underlying sexual arousal and orgasm in humans. World J Urol.

[15] Qamar M, Sauerbier A, Politis M, et al (2017) Presynaptic dopaminergic terminal imaging and non-motor symptoms assessment of Parkinson’s disease: evidence for dopaminergic basis? npj Parkinson’s Disease 3:5. 10.1038/s41531-016-0006-9 10.1038/s41531-016-0006-9 PMC5445592 28649605

[16] van Deursen DN, van den Heuvel OA, Booij J (2020). Autonomic failure in Parkinson’s disease is associated with striatal dopamine deficiencies. J Neurol.

[17] Kim R, Jun J-S (2019). Association of autonomic symptoms with presynaptic striatal dopamine depletion in drug-naive Parkinson’s disease: an analysis of the PPMI data. Auton Neurosci.

[18] Park SB, Kwon K-Y, Lee J-Y (2019). Lack of association between dopamine transporter loss and non-motor symptoms in patients with Parkinson’s disease: a detailed PET analysis of 12 striatal subregions. Neurol Sci.

[19] Hughes AJ, Daniel SE, Kilford L, Lees AJ (1992). Accuracy of clinical diagnosis of idiopathic Parkinson’s disease: a clinico-pathological study of 100 cases. J Neurol Neurosurg Psychiatry.

[20] Movement Disorder Society Task Force on Rating Scales for Parkinson’s Disease (2003). The Unified Parkinson’s Disease Rating Scale (UPDRS): status and recommendations. Mov Disord.

[21] Goetz CG, Poewe W, Rascol O (2004). Movement Disorder Society Task Force report on the Hoehn and Yahr staging scale: status and recommendations. Mov Disord.

[22] Kägi G, Bhatia KP, Tolosa E (2010). The role of DAT-SPECT in movement disorders. J Neurol Neurosurg Psychiatry.

[23] Stiasny-Kolster K, Mayer G, Schäfer S (2007). The REM sleep behavior disorder screening questionnaire–a new diagnostic instrument. Mov Disord.

[24] Nomura T, Inoue Y, Kagimura T (2011). Utility of the REM sleep behavior disorder screening questionnaire (RBDSQ) in Parkinson’s disease patients. Sleep Med.

[25] Folstein MF, Folstein SE, McHugh PR (1975). “Mini-mental state”. A practical method for grading the cognitive state of patients for the clinician. J Psychiatr Res.

[26] McColgan P, Evans JR, Breen DP (2012). Addenbrooke’s Cognitive Examination-Revised for mild cognitive impairment in Parkinson’s disease. Mov Disord.

[27] Leentjens AF, Verhey FR, Luijckx GJ, Troost J (2000). The validity of the Beck Depression Inventory as a screening and diagnostic instrument for depression in patients with Parkinson’s disease. Mov Disord.

[28] Leentjens AFG, Dujardin K, Marsh L (2008). Anxiety rating scales in Parkinson’s disease: critique and recommendations. Mov Disord.

[29] Chaudhuri KR, Martinez-Martin P, Brown RG (2007). The metric properties of a novel non-motor symptoms scale for Parkinson’s disease: results from an international pilot study. Mov Disord.

[30] Nobili F, Naseri M, De Carli F (2013). Automatic semi-quantification of [123I]FP-CIT SPECT scans in healthy volunteers using BasGan version 2: results from the ENC-DAT database. Eur J Nucl Med Mol Imaging.

[31] Skanjeti A, Angusti T, Iudicello M (2014). Assessing the accuracy and reproducibility of computer-assisted analysis of (123) I-FP-CIT SPECT using BasGan (V2). J Neuroimaging.

[32] Robinson BW, Mishkin M (1968). Penile erection evoked from forebrain structures in Macaca mulatta. Arch Neurol.

[33] Seok J-W, Sohn J-H, Cheong C (2016). Neural substrates of sexual arousal in heterosexual males: event-related fMRI investigation. J Physiol Anthropol.

[34] Votinov M, Goerlich KS, Puiu AA (2021). Brain structure changes associated with sexual orientation. Sci Rep.

[35] Erro R, Pappatà S, Amboni M (2012). Anxiety is associated with striatal dopamine transporter availability in newly diagnosed untreated Parkinson’s disease patients. Parkinsonism Relat Disord.

[36] Vriend C, Raijmakers P, Veltman DJ (2014). Depressive symptoms in Parkinson’s disease are related to reduced [123I]FP-CIT binding in the caudate nucleus. J Neurol Neurosurg Psychiatry.

[37] Santangelo G, Vitale C, Picillo M (2015). Apathy and striatal dopamine transporter levels in de-novo, untreated Parkinson’s disease patients. Parkinsonism Relat Disord.

[38] Spiegel J, Hellwig D, Samnick S (2007). Striatal FP-CIT uptake differs in the subtypes of early Parkinson’s disease. J Neural Transm (Vienna).

[39] Moccia M, Pappatà S, Picillo M (2014). Dopamine transporter availability in motor subtypes of de novo drug-naïve Parkinson’s disease. J Neurol.

[40] Voon V, Napier TC, Frank MJ (2017). Impulse control disorders and levodopa-induced dyskinesias in Parkinson’s disease: an update. Lancet Neurol.

[41] Graf H, Malejko K, Metzger C (2019). Serotonergic, dopaminergic, and noradrenergic modulation of erotic stimulus processing in the male human brain. JCM.

